# P-728. Chlamydia and Gonorrhea Infections Are Associated with Hypertensive Disorders of Pregnancy

**DOI:** 10.1093/ofid/ofaf695.939

**Published:** 2026-01-11

**Authors:** Kaitlyn Broderick, Ellen Caniglia, Mary Regina Boland, Alisa J Stephens Shields

**Affiliations:** University of Pennsylvania Perelman School of Medicine, Decatur, Georgia; University of Pennsylvania, Philadelphia, Pennsylvania; Saint Vincent College, Latrobe, Pennsylvania; University of Pennsylvania, Philadelphia, Pennsylvania

## Abstract

**Background:**

Hypertensive disorders of pregnancy (HDP) are a leading cause of maternal morbidity and mortality in the U.S. Bacterial sexually transmitted infections (STIs), such as *Chlamydia trachomatis* and *Neisseria gonorrhoeae*, may contribute to HDP through infection-induced inflammation affecting placental development. Evidence for this association remains limited, particularly in urban U.S. populations with routine STI screening.

**Figure:**
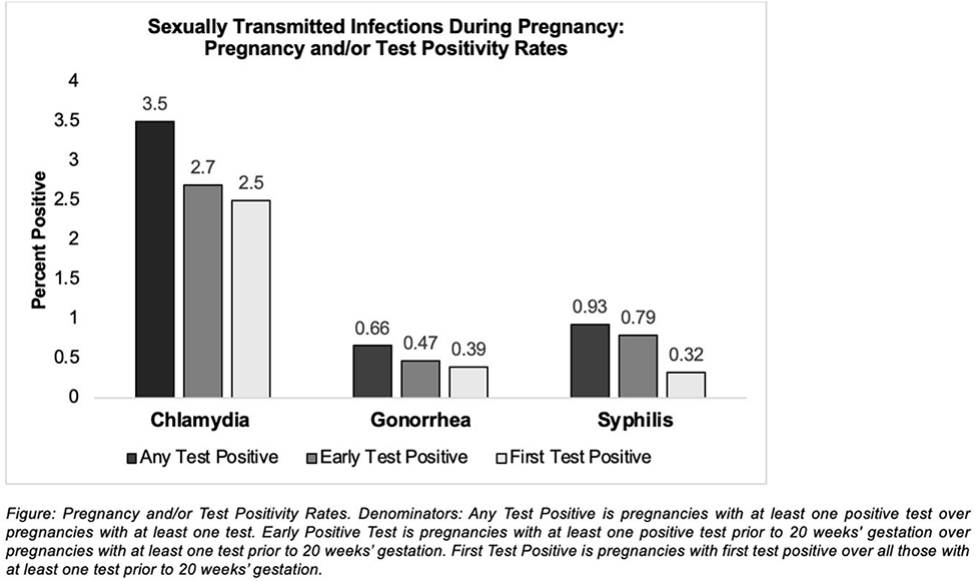

**Figure:**
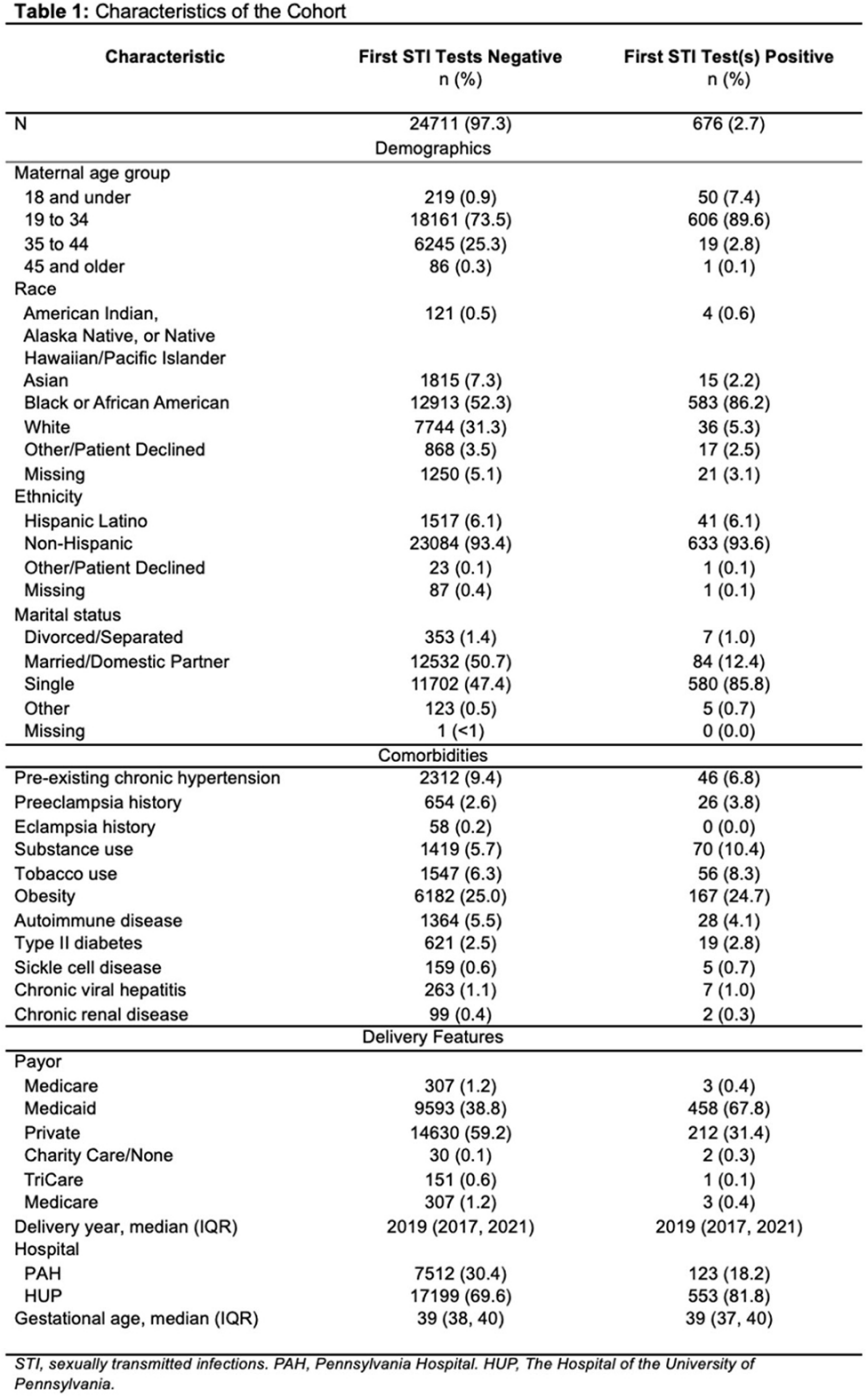

**Methods:**

We performed a retrospective cohort study using electronic medical records from two Penn Medicine hospitals in Philadelphia. We identified singleton deliveries from 2010–2022 with chlamydia/gonorrhea testing before 20 weeks gestation. The primary exposure was a positive initial test for chlamydia and/or gonorrhea. The primary outcome was HDP, defined as a composite of gestational hypertension, preeclampsia, eclampsia, or superimposed preeclampsia, based on ICD-9/10 codes. Multivariable logistic regression with generalized estimating equations adjusted for sociodemographic and clinical covariates.

**Figure:**
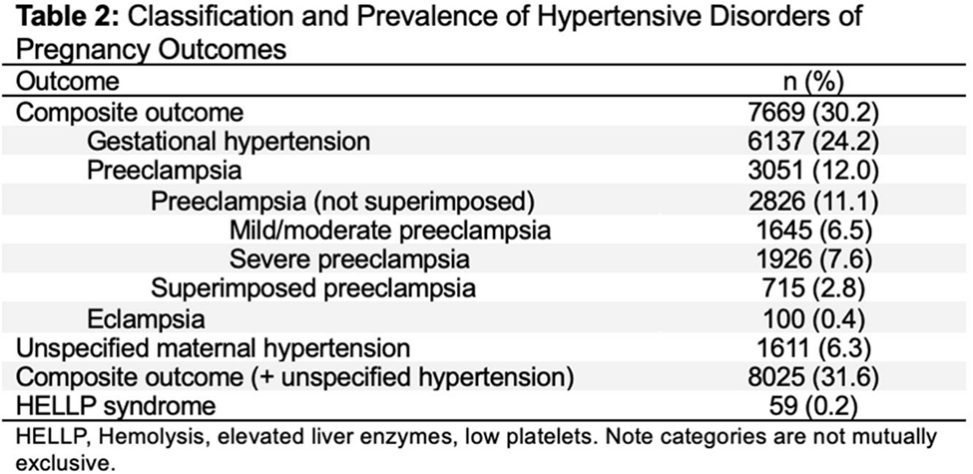

**Figure:**
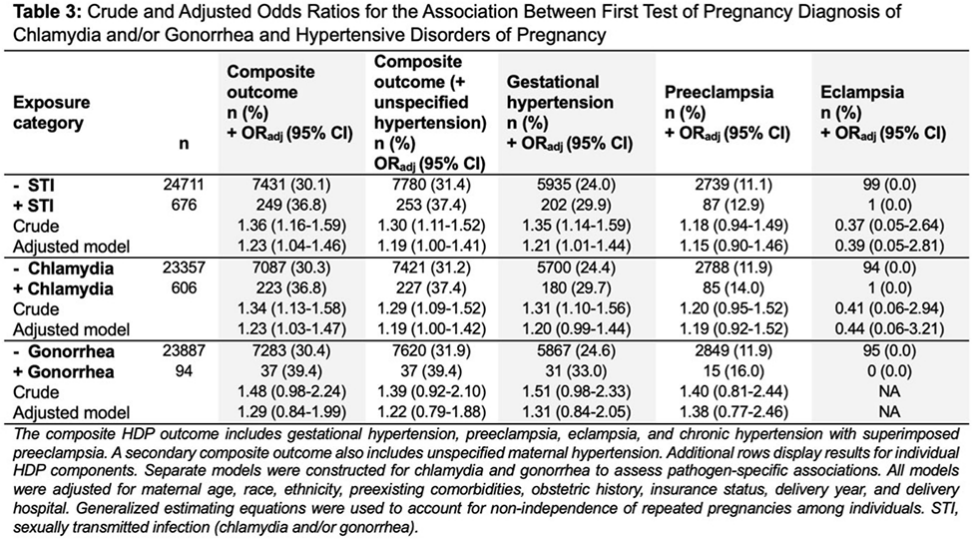

**Results:**

Among 25,387 pregnancies, 2.7% had a positive initial STI test. HDP occurred in 31.6% of the cohort. A positive initial STI test was associated with increased odds of HDP (adjusted odds ratio [aOR]: 1.23; 95% CI: 1.04–1.46). The association was driven largely by gestational hypertension (aOR: 1.21; 95% CI: 1.01–1.44). Chlamydia alone was significantly associated with HDP (aOR: 1.23; 95% CI: 1.03–1.47). Gonorrhea showed a similar but non-significant trend (aOR: 1.29; 95% CI: 0.84–1.99). No associations were observed for preterm birth or severe maternal morbidity. Findings were consistent in sensitivity analyses.

**Conclusion:**

Early pregnancy diagnosis of chlamydia and possibly gonorrhea is associated with increased risk of HDP. These findings support further research into the role of maternal infection in HDP pathogenesis and may inform STI screening policies in pregnancy.

**Disclosures:**

All Authors: No reported disclosures

